# SPOUSAL HEALTH SHOCKS AND INDIVIDUALS’ LABOR PARTICIPATION AND RETIREMENT DECISION: CAUSAL EVIDENCE FROM THE US

**DOI:** 10.1093/geroni/igae098.0604

**Published:** 2024-12-31

**Authors:** Meiyi Li

**Affiliations:** University of Wisconsin-Madison, Madison, Wisconsin, United States

## Abstract

Health decline is a dyadic process that elicits labor adjustments from both individuals and their intimate partners. Despite increasing attention to the spillover partner effects, empirical evidence is still mixed and the causal nature remains unclear. Drawing upon the nationally representative data from the Health and Retirement Study (1992-2018), the current study aims to investigate (a) What are the causal impacts of spousal health shocks (cancer diagnosis, strokes, and heart attacks) on individuals’ employment, retirement behaviors, and retirement expectations? (b) Are there any gender differences? Matching methods are combined with a difference-in-differences design to examine the causal impacts of spousal health shocks. Results suggest spousal health shocks reduce the probability of working for pay and increase the probability of retirement. As for retirement decisions, spousal health shocks reduce older adults’ expected probability of working after 65. Additionally, results do not show significant gender differences in labor outcomes. Overall, the current study provides important causal evidence on older adults’ labor responses to spousal health shocks. It also informs dyadic interventions to help both people with health decline and their partners maintain economic and social well-being in later life.

